# Epidemiological indicators of Chagas disease in the metropolitan region of Salvador, Bahia, Brazil

**DOI:** 10.1590/0037-8682-0185-2022

**Published:** 2023-02-20

**Authors:** Fernanda Cardoso Lanza, Gilmar Ribeiro-Jr, Diego Lopes Paim Miranda, Fred Luciano Neves Santos, Cristiane Medeiros Moraes de Carvalho, Gabriel Muricy Cunha, Ianei de Oliveira Carneiro, Renato Barbosa Reis, José Maurício Albuquerque Cunha, Cristiane Wanderley Cardoso, Jorgana Fernanda de Souza Soares, Fernando Luiz Vieira de Araújo, Mitermayer Galvão Reis

**Affiliations:** 1 Fundação Oswaldo Cruz, Instituto Gonçalo Moniz, Laboratório de Patologia e Biologia Molecular, Salvador, BA, Brasil.; 2 Universidade Federal da Bahia, Faculdade de Medicina da Bahia, Salvador, BA, Brasil.; 3 Secretaria da Saúde do Estado da Bahia, Superintendência de Vigilância em Saúde, Diretoria de Vigilância Epidemiológica, Salvador, BA, Brasil.; 4 Universidade Salvador, Salvador, BA, Brasil.; 5 Centro Universitário Estácio de Sá, Campus Gilberto Gil, Salvador, BA, Brasil.; 6 Secretaria Municipal de Saúde de Salvador, Salvador, BA, Brasil.; 7 Fundação de Hematologia e Hemoterapia da Bahia, Salvador, BA, Brasil.; 8 Yale University, Yale School of Public Health, Department of Epidemiology of Microbial Diseases, New Haven, Connecticut, USA.

**Keywords:** Trypanosoma cruzi, Public health, Epidemiological surveillance

## Abstract

**Background:**

Chagas disease (CD) is caused by *Trypanosoma cruzi* and transmitted by triatomines. Historical information from the 20^th^ century demonstrates *T. cruzi* records in the metropolitan region of Salvador (MRS), the third largest urban agglomeration in the Brazilian Northeast and the eighth largest in Brazil, an area with intense migratory activity from CD-endemic regions. Therefore, this study aimed to evaluate CD indicators (prevalence and mortality) in the MRS.

**Methods:**

A mixed ecological and descriptive study was conducted using secondary data. We analyzed data from 2008 to 2015: deaths due to CD, self-reported cases of CD, and blood donors that were non-negative for *T. cruzi* infection.

**Results:**

São Francisco do Conde was one of the municipalities with the highest mortality rates due to CD. The seroprevalence rates varied by year and municipality; those with the highest values were 2008: Vera Cruz, 2009: Mata de São João, 2010: Dias D'Ávila, 2011 and 2015: São Francisco do Conde, 2012: São Sebastião do Passé, and 2013 and 2014: Pojuca. Spatial correlations between the municipalities were not detected.

**Conclusions:**

We conclude that CD is present in the MRS. The indicators analyzed in the MRS are below-state-level data. Given the importance of indicator analysis for the surveillance and control of CD at the state and national levels, it is important to strengthen the surveillance program at the municipal level, including the regions classified as low risk for *T. cruzi* vector transmission.

## INTRODUCTION

Chagas disease (CD) is caused by the flagellated protozoan parasite *Trypanosoma cruzi* (Chagas, 1909), transmitted by the insect vector, triatomine bugs^1^. It is estimated that 6-7 million people are affected by CD worldwide (mainly in Latin American countries)^2^. In Brazil, 1.9-4.6 million people live with the disease; specifically, Bahia has an average of 624 deaths per year, the fourth state with the highest mortality rate from 2010 to 2019^3^ ^,^ ^4^. In Bahia, there are many records of people with chronic CD, and in the last 10 years, two acute cases have been confirmed only according to records in information systems. Occurrence of CD is probably underreported^4^.

Historically, the prevalence *T. cruzi* infection was higher in rural areas. However, owing to migratory movements, patients with CD have spread to urban areas. Therefore, most infected people are believed to live in urban areas^6^ ^,^ ^7^, which requires epidemiological surveillance to understand the extent of the problem and to plan effective prevention and control measures for CD, as well as specialized medical care and treatment for infected patients living in these areas.

Historical records indicate that *T. cruzi* has been present in the metropolitan region of Salvador (MRS), particularly in the municipality of Mata de São João since the early 20^th^ century^5^. In the early 1970s, in one of the last research projects in the city of Salvador, 149 specimens of *Panstrongylus megistus* and 452 specimens of *Triatoma rubrofasciata* were found in the historic center (Pelourinho). Of these, 16% were infected by *T. cruzi* ^7^. In recent years, triatomines infected with *T. cruzi* have been identified visiting households in urban areas in fragments of the Atlantic Forest biome in the municipality of Salvador. These studies demonstrated the frequent occurrence of *Triatoma tibiamaculata* infected with *T. cruzi* in houses and apartments near deforested areas in several neighborhoods of the city^8^ ^,^ ^9^. However, there is little information about triatomines in the municipal databases of the health surveillance system in the MRS. Therefore, we decided to study CD in the municipalities of MRS, considering other parameters to provide information to support health managers on the importance of entomological surveillance.

Surveillance activities in Brazil are usually triggered by acute CD cases reported in the Notifiable Diseases Information System (SINAN)^10^, although the number of cases reported in this system has been underreported^11^. Therefore, it is necessary to use other data sources, such as medical assistance, to support public health interventions.

Follow-up of patients with CD in the MRS should be mainly in the primary care of the Public Health System. Severe cases of acute or reactive disease and decompensated chronic cases should be referred to other levels of care and, if necessary, specialized hospitals^12^. Due to diagnostic difficulties in the acute phase of the disease, many cases progress to the chronic form, whose clinical manifestations may be cardiac, digestive, or mixed^10^. Among the neglected tropical diseases in Brazil, CD had the highest proportion of global disease burden in 2016 (29.8% of the total)^13^.

The results of serological screening of blood donors provide important information for CD. This initiative was launched in 1991 by the Southern Cone Initiative with the goal of reducing and eliminating blood transfusion transmission through serological screening of donors from blood banking networks^14^. Blood transfusion transmission is considered the second most common cause of CD in non-endemic industrialized countries, particularly in countries receiving immigrants from Latin America^15^. The prevalence of *T. cruzi* infection in blood donors is a powerful epidemiological indicator and can be used as an indicator of CD transfusion risk and transmission in an area^14^ ^,^ ^16^. From 2008 to 2018, the Foundation for Hematology and Hemotherapy of the State of Bahia (HEMOBA) presented 500,256 blood donors. Of these donors, 3,084 (0.62%) tested positive for CD. It was observed that 1,108 (35.9%) of the screened cases corresponded to people living in Salvador^4^.

Primary health care data on the follow-up of patients with CD, serologic testing of blood donors, and mortality from CD may be useful information to identify priority areas for public health interventions. Therefore, this study aimed to evaluate CD indicators (prevalence and mortality) in the municipalities of MRS.

## METHODS

### Study area

This study evaluated only CD indicators from the MRS, which includes the capital of the state of Bahia (Salvador) and 12 municipalities: Camaçari, Candeias, Dias D'Ávila, Itaparica, Lauro de Freitas, Madre de Deus, Mata de São João, Pojuca, São Francisco do Conde, São Sebastião do Passé, Simões Filho, and Vera Cruz^17^. According to the Brazilian Institute of Geography and Statistics (IBGE), MRS is the second largest metropolitan region in northeastern Brazil and even in the whole country, with an area of 4,375,123 km² and a population of 3,929,209 inhabitants. The Gross Domestic Product (GDP) is R$ 122,780,193.11, and the *per capita* income is R$ 31,485.87^18^.

### Epidemiological data

We analyzed data from 2008 to 2015 using the following variables: (1) deaths due to CD, (2) self-reported cases of CD registered in the Primary Care Information System (SIAB), and (3) blood donors registered as non-negative for *T. cruzi* infection in the serological screening performed by the HEMOBA Foundation. The samples considered non-negative by the HEMOBA Foundation were those with reactive results for *T. cruzi* infection. High-sensitivity tests are mandatory for screening for CD. From 1991 to 2015, the HEMOBA used *anti-T. cruzi* and enzyme-linked immunosorbent assay (ELISA). In February 2015, the HEMOBA began to perform serological screening through chemiluminescence tests with IgG and IgM antibodies. The same test was used in the second screening, and in case of positivity, donors were sent to a referral unit^9^. Data were provided by the Department of Epidemiological Surveillance of the Ministry of Health of the State of Bahia (DIVEP/SESAB) and HEMOBA Foundation.

### Data analysis and geoprocessing

Data were analyzed considering the prevalence or mortality of CD per 100,000 inhabitants in each municipality. The rate per 100,000 inhabitants was obtained considering the population of each municipality yearly, according to the IBGE census, through the Basic Care Information and Management website (https://egestorab.saude.gov.br/paginas/acessoPublico/relatorios/relHistoricoCoberturaAB.xhtml)^18^. For geoprocessing, the geographic information system of QGIS ^™^ software version 3.20.3 was used to analyze and determine the information patterns. Shapefiles containing the geographic boundaries of the municipalities of MRS were obtained from the IBGE database (https://downloads.ibge.gov.br)^20^. The spatial unit used for georeferencing was the name of the municipality or IBGE geocode. The GeoDa^®^ (Center for Spatial Data Science, University of Chicago) tool was used to perform a spatial autocorrelation analysis with the values of the prevalence coefficients of the epidemiological variables. Through a spatial statistical analysis considering the average values of the prevalence coefficients of the three previously presented CD indicators, GeoDa software was used to test for spatial autocorrelation through the analysis of the Moran’s index during the study period. 

### Ethics statement

All databases used in this study are available in the public domain and do not allow the identification of individuals. In 2016, a new resolution published by the Brazilian National Health Council abated the need to seek approval from any Institutional Review Board for studies using publicly available secondary data that did not provide identifiable information (http://conselho.saude.gov.br/resolucoes/2016/reso510.pdf).

## RESULTS

### Death due to Chagas disease

**FIGURE 1:**
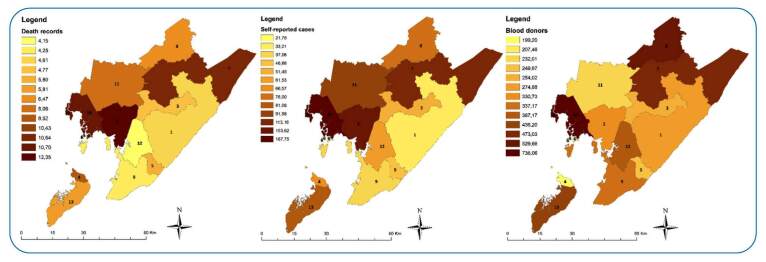
Result of geostatistical analysis based on data described in deaths due to Chagas disease; self-reported prevalence coefficient (PC) Chagas disease/100,000 inhabitants of Chagas disease registered in SIAB, and seroprevalence coefficient (SC) for T. cruzi infections/100,000 blood donors from the HEMOBA Foundation. Legend: (1) = Camaçari; (2) = Candeias; (3) = Dias D’Ávila; (4) = Itaparica; (5) = Lauro de Feitas; (6) = Madre de Deus; (7) = Mata de São João; (8) = Pojuca; (9) = Salvador; (10) = São Francisco do Conde; (11) = São Sebastião do Passé; (12) = Simões Filho; (13) = Vera Cruz.

A total of 1,517 deaths due to CD were recorded between 2008 and 2015. The municipalities with the highest mortality rates from CD are listed in Table 1. In 2008, 2009, 2010, and 2013, São Francisco do Conde remained one of the municipalities with the highest mortality rate due to CD, with 23.5, 16, 15.8, and 17.5/100,000 inhabitants, respectively. Other municipalities that also had high mortality rates during the study period were Mata de São João and Candeias (Figure 1).

**TABLE 1:** Cause-specific mortality rate (CSMR) due to Chagas disease/100,000 inhabitants in municipalities of the metropolitan region of Salvador, 2008-2015.

	Year																							
Municipality Residence RMS	2008			2009			2010			2011			2012			2013			2014			2015		
	Cases	Population	CSMR	Cases	Population	CSMR	Cases	Population	CSMR	Cases	Population	CSMR	Cases	Population	CSMR	Cases	Population	CSMR	Cases	Population	CSMR	Cases	Population	CSMR
Camaçari	12	220,495	5.4	11	227,955	4.8	18	234,558	7.7	18	242,970	7.4	11	249,206	4.4	15	255,238	5.9	11	275,575	4.0	10	281,413	3.6
Candeias	13	78,618	16.5	8	81,306	9.8	13	81,699	15.9	14	83,158	16.8	12	83,647	14.3	16	84,121	19.0	8	89,419	8.9	9	88,308	10.2
Dias d'Ávila	8	53,821	14.9	3	56,600	5.3	2	57,708	3.5	4	66,440	6.0	2	68,060	2.9	4	69,628	5.7	4	75,103	5.3	4	76,624	5.2
Itaparica	0	19,897	0.0	0	20,641	0.0	1	20,796	4.8	1	20,725	4.8	3	20,862	14.4	3	20,994	14.3	2	22,329	9.0	2	22,476	8.9
Lauro de Freitas	8	144,492	5.5	13	153,016	8.5	8	156,936	5.1	17	163,449	10.4	12	167,308	7.2	12	171,042	7.0	10	184,383	5.4	9	188,013	4.8
Madre de Deus	0	15,432	0.0	2	16,354	12.2	0	16,783	0.0	0	17,376	0.0	2	17,786	11.2	2	18,183	11.0	1	19,600	5.1	2	19,985	10.0
Mata de São João	7	37,201	18.8	4	38,962	10.3	5	39,585	12.6	7	40,183	17.4	4	40,866	9.8	4	41,527	9.6	4	44,538	9.0	3	45,194	6.6
Pojuca	0	30,221	0.0	2	31,687	6.3	6	32,225	18.6	4	33,066	12.1	1	33,595	3.0	1	34,106	2.9	3	36,551	8.2	2	37,061	5.4
Salvador	132	2,892,625	4.6	108	2,948,733	3.7	129	2,998,056	4.3	125	2,675,656	4.7	125	2,693,605	4.6	113	2,710,968	4.2	132	2,883,682	4.6	133	2,902,927	4.6
São Francisco do Conde	7	29,829	23.5	5	31,219	16.0	5	31,699	15.8	3	33,183	9.0	3	33,713	8.9	6	34,226	17.5	3	36,677	8.2	4	38,838	10.3
São Sebastião do Passé	3	40,321	7.4	3	41,624	7.2	4	41,758	9.6	4	42,153	9.5	1	42,321	2.4	5	42,485	11.8	4	45,090	8.9	5	45,292	11.0
Simões Filho	7	109,269	6.4	8	114,649	7.0	5	116,662	4.3	2	118,047	1.7	6	119,759	5.0	1	121,416	0.8	8	129,964	6.2	3	131,630	2.3
Vera Cruz	2	35,060	5.7	2	36,843	5.4	2	37,539	5.3	4	37,567	10.6	1	38,167	2.6	1	38,748	2.6	3	41,524	7.2	3	42,103	7.1

**Source:** SESAB/SUVISA/DIS/Mortality Information System The data are updated on April 11, 2018. **CSMR:** Cause specific mortality rate per 100.000 population.

### Self-reported prevalence coefficient Chagas disease/100,000 inhabitants of Chagas disease registered in the primary care information system

Prevalence per 100,000 inhabitants for MRS municipalities can be found in Table 2. From 2008 to 2014, Candeias had the highest prevalence per 100,000 inhabitants (302.8, 340.9, 262.2, 260.6, 231.9, 230.9, 226.1 cases per 100,000 inhabitants), followed by São Francisco do Conde (167.6, 157, 132.5, 211, 243.2, 364.4, 220.3, and 198.3 cases per 100,000 inhabitants from 2008 to 2015) (Figure 1). 

**TABLE 2:** Self-reported prevalence coefficient (PC) of Chagas disease/100,000 inhabitants among the population covered by the primary health care of in the municipalities of the metropolitan region of Salvador, 2008-2015**.**

	Year																							
Municipality Residence RMS	2008			2009			2010			2011			2012			2013			2014			2015		
	Cases	Population	PC	Cases	Population	PC	Cases	Population	PC	Cases	Population	PC	Cases	Population	PC	Cases	Population	PC	Cases	Population	PC	Cases	Population	PC
Camaçari	63	164,550	38.3	73	136,350	53.5	88	196,650	44.7	107	184,650	57.9	109	169,200	64.4	112	195,300	57.3	112	214,800	52.1	112	198,900	56.3
Candeias	137	45,240	302.8	145	42,540	340.9	142	54,150	262.2	139	53,340	260.6	134	57,780	231.9	131	56,730	230.9	132	58,380	226.1	0	54,480	0.0
Dias d'Ávila	43	42,000	102.4	41	34,500	118.8	48	34,500	139.1	38	26,490	143.5	40	30,150	132.7	38	51,750	73.4	38	56,850	66.8	0	54,750	0.0
Itaparica	14	17,250	81.2	14	20,641	67.8	16	20,700	77.3	16	20,700	77.3	16	20,862	76.7	15	20,700	72.5	15	22,329	67.2	0	22,476	0.0
Lauro de Freitas	98	100,830	97.2	102	84,840	120.2	96	78,390	122.5	86	93,210	92.3	90	117,960	76.3	86	123,750	69.5	86	137,490	62.6	86	125,100	68.7
Madre de Deus	7	15,432	45.4	6	16,354	36.7	5	16,783	29.8	5	17,250	29.0	4	17,250	23.2	3	17,250	17.4	3	17,250	17.4	3	17,250	17.4
Mata de São João	48	37,201	129.0	50	35,280	141.7	53	39,585	133.9	55	40,183	136.9	55	40,866	134.6	52	41,527	125.2	51	44,538	114.5	56	45,194	123.9
Pojuca	23	30,221	76.1	24	31,687	75.7	31	32,225	96.2	33	33,066	99.8	32	33,595	95.3	30	34,106	88.0	29	36,551	79.3	29	37,061	78.2
Salvador	1214	796,590	152.4	1187	815,400	145.6	1213	942,930	128.6	1096	834,000	131.4	1078	711,390	151.5	1021	973,680	104.9	945	1,026,630	92.0	962	1,074,180	89.6
São Francisco do Conde	50	29,829	167.6	49	31,219	157.0	42	31,699	132.5	70	33,183	211.0	82	33,713	243.2	88	24,150	364.4	76	34,500	220.3	77	38,838	198.3
São Sebastião do Passé	51	40,321	126.5	42	41,624	100.9	38	41,758	91.0	41	41,400	99.0	39	41,400	94.2	41	42,485	96.5	42	45,090	93.1	0	44,400	0.0
Simões Filho	109	52,950	205.9	105	59,490	176.5	93	50,250	185.1	99	55,650	177.9	56	65,100	86.0	87	68,100	127.8	79	53,100	148.8	35	53,100	65.9
Vera Cruz	35	34,500	101.4	35	36,843	95.0	35	37,539	93.2	35	27,600	126.8	35	38,167	91.7	35	38,748	90.3	35	41,400	84.5	35	37,950	92.2

**Source:** Ministry of Health - Primary Care Information System - SIAB. **PC:** prevalence coefficient per 100.000 population.

### Seroprevalence coefficient for *T. cruzi* infection/100,000 HEMOBA Foundation blood donors

Between 2008 and 2015, the total number of blood donors residing in the MRS was 288,281 (Table 3). Of these, 960 (0.003%) donors were non-negative for *T. cruzi* infection. In 2008, Vera Cruz had the highest seroprevalence coefficient (4,545.5 per 100,000 inhabitants); in 2009, Mata de São João (2,531.6 per 100,000 inhabitants); in 2010, Dias D’Ávila (591.7/100,000 inhabitants); in 2011 and 2015, São Francisco do Conde (1,282.1 and 2,479.3 per 100,000 inhabitants, respectively); in 2012, São Sebastião do Passé (1,047.1 per 100,000 inhabitants); and in 2013 and 2014, Pojuca (833.3 and 704.2/100,000 inhabitants, respectively) (Figure 1).

**TABLE 3:** Seroprevalence coefficient (SC) for T. cruzi infections/100,000 blood donors in the municipalities of the metropolitan region of Salvador, 2008-2015.

	Year																							
Municipality Residence RMS	2008			2009			2010			2011			2012			2013			2014			2015		
	Cases	Population	SC	Cases	Population	SC	Cases	Population	SC	Cases	Population	SC	Cases	Population	SC	Cases	Population	SC	Cases	Population	SC	Cases	Population	SC
Dias d'Ávila	1	174	574.7	1	310	322.6	1	169	591.7	1	330	303.0	0	264	0.0	1	366	273.2	1	380	263.2	0	369	0.0
Itaparica	0	32	0.0	1	64	1,562.5	0	61	0.0	0	70	0.0	0	60	0.0	0	83	0.0	0	64	0.0	0	68	0.0
Lauro de Freitas	5	1,188	420.9	1	1,268	78.9	0	1,354	0.0	1	1,616	61.9	4	1,519	263.3	7	1,422	492.3	5	1,715	291.5	7	1,919	364.8
Madre de Deus	0	122	0.0	0	82	0.0	0	133	0.0	0	135	0.0	2	194	1,030.9	0	254	0.0	0	175	0.0	1	351	284.9
Mata de São João	0	80	0.0	2	79	2,531.6	0	75	0.0	1	112	892.9	0	171	0.0	1	183	546.4	0	149	0.0	1	208	480.8
Pojuca	1	100	1,000.0	2	85	2,352.9	0	76	0.0	0	98	0.0	0	179	0.0	1	120	833.3	1	142	704.2	0	144	0.0
Salvador	121	25,671	471.3	47	29,378	160.0	56	30,087	186.1	71	30,785	230.6	160	32,510	492.2	95	30,419	312.3	143	30,484	469.1	124	32,971	376.1
São Francisco do Conde	1	87	1,149.4	0	95	0.0	0	200	0.0	1	78	1,282.1	1	118	847.5	1	133	751.9	0	119	0.0	3	121	2,479.3
São Sebastião do Passé	1	229	436.7	0	130	0.0	0	116	0.0	0	95	0.0	2	191	1,047.1	0	202	0.0	0	161	0.0	0	169	0.0
Simões Filho	4	950	421.1	3	917	327.2	4	1,067	374.9	2	1,140	175.4	7	1,181	592.7	4	975	410.3	4	924	432.9	4	1,111	360.0
Vera Cruz	3	66	4,545.5	0	89	0.0	0	103	0.0	0	118	0.0	0	170	0.0	0	125	0.0	0	110	0.0	1	136	735.3

**Source:** HEMOBA Foundation.

We did not find statistical evidence of spatial autocorrelation between municipalities when analyzing the three variables cited. The Moran's index value was 0.064, and the *P*-value in the pseudo-significance test was 0.42, confirming spatial independence between the municipalities. As shown in Figure 1, there are similarities between deaths due to CD and self-reported cases in many regions and even among blood donors, although, proportionally, infection in blood banks is higher. The municipalities with the highest evidence of CD, according to the variables, were São Francisco do Conde, Mata de São João, and Candeias.

## DISCUSSION

Based on the analysis of the three variables investigated in the present study, the municipalities of São Francisco do Conde, Mata de São João, and Candeias showed higher rates of CD, indicating that there is still a risk of transmission in some areas of the MRS in Bahia.

Deaths due to CD were observed in all municipalities of MRS. Notably, approximately 20.8% of the deaths occurred in a different municipality than the one where the person lived^22^. This could be related to the patient demand for better medical care and hospital infrastructure, which could overwhelm public health administration planning^10^. In addition, there are no official guidelines for recording deaths due to CD, as there are other endemic diseases. This is also an issue raised by the Ministry of Health and Death Review Service. A clinical assessment by a medical team defines the underlying cause of death, and health workers can use the International Statistical Classification of Diseases and Related Health Problems (ICD): B57.0, B57.1, B57.2, B57.3, B57.4, and B57.523.

In all municipalities of MRS, there were records of self-reported cases of CD. A section on self-reported diseases or conditions can be completed using SIAB application forms. It is possible to register a CD. In this case, community health providers should not require proof of diagnosis^4^, which indicates the need to improve access to diagnosis, specific treatment, and follow-up for patients with CD. In addition, it is not possible to determine the incidence, but only the prevalence, because it is not possible to define whether the cases registered each year are new or recurrent.

Regarding serological screening at the HEMOBA Foundation, there were blood donors who were non-negative for *T. cruzi* from all municipalities of the MRS, which were recorded as either city of birth and/or residence. The prevalence of *T. cruzi* infection in blood donors may be an important epidemiological indicator, consolidating its position as a transfusion risk marker for CD and measuring the degree of transmission of the disease in a region^14^ ^,^ ^19^ ^-^ ^24^. In Brazil, the incidence of serological reactions resulting from reagent samples for *T. cruzi* has decreased significantly over the years because of the positive results of triatomine control and improvement in the quality of life of the population in formerly endemic areas^25^ ^,^ ^26^. The positive serology for *T. cruzi* from 1987 to 1994 was 0.77-2.22% in Bahia and 0.69-0.88% in northeastern Brazil. The seropositivity in Brazil from 1988 to 1990 was 0.97%, with a decrease in the following years; 0.7-1.1% in 1991, 0.7% in 1993, was, and 0.75% in 1994. From 2008 to 2018, the seroprevalence was 0.62%^14^ ^,^ ^19^. Evaluation of the distribution of reactivity among the municipalities of MRS showed that Salvador had the most reactive serologies compared with the other municipalities. This could be a consequence of migration from endemic areas to the city of Salvador, either within or outside Bahia.

This study has methodological limitations common to studies based on secondary data, particularly regarding the underreporting of deaths due to CD. In addition, despite all efforts to include entomological indicators, this was not possible because triatomines were not recorded in most municipalities. It is important to increase health communication for CD on social media and keep health professionals informed to make them aware of the suspicion, diagnosis, treatment, and follow-up of patients with CD. Thus, professionals can monitor the disease and collect data for surveillance systems.

The data of this study suggest that more attention should be paid to CD monitoring systems and that surveillance of CD is necessary for the municipalities of MRS and others^8^ ^,^ ^9^. Conducting studies to identify social, economic, and environmental factors involved in the epidemiological context of CD will help develop measures to control the infection and improve the documentation of cases in these localities. In addition, new methods should be applied, such as analyzing and estimating the underreporting of cases/deaths, to update the risk classification for CD in the MRS.
